# Evaluation of Four Humanized NOD-Derived Mouse Models for Dengue Virus-2 Infection

**DOI:** 10.3390/pathogens13080639

**Published:** 2024-07-30

**Authors:** Hernando Gutierrez-Barbosa, Sandra Medina-Moreno, Federico Perdomo-Celis, Harry Davis, Joel V. Chua, Juan C. Zapata

**Affiliations:** 1 Institute of Human Virology, University of Maryland School of Medicine, Baltimore, MD 21201, USA; hector.gutierrezb@udea.edu.co (H.G.-B.); smedinamoreno@gmail.com (S.M.-M.); jchua@ihv.umaryland.edu (J.V.C.); 2 Facultad de Biología, Universidad de Antioquia, Bogotá 050010, Colombia; 3 Instituto de Genética Humana, Facultad de Medicina, Pontificia Universidad Javeriana, Bogotá 110231, Colombia; perdomo_federico@javeriana.edu.co

**Keywords:** humanized mice, dengue, NSG, EXL, SGM3, NCG, CD34^+^, PBMCs

## Abstract

Dengue is a significant public health problem with no specific viral treatment. One of the main challenges in studying dengue is the lack of adequate animal models recapitulating human immune responses. Most studies on humanized mice use NOD-scid IL2R gamma null (NSG) mice, which exhibit poor hematopoiesis for some cell populations. This study compares three humanized (hu) NOD-derived mouse models for dengue virus-2 (DENV-2) infection in the context of human cytokine expression. Three mouse strains (hu-NSG, hu-EXL, and hu-SGM3) received xenotransplants of human CD34+ fetal cord blood cells from a single donor, and one mouse strain received human peripheral blood mononuclear cells (hu-SGM3-PBMCs). All models exhibited infectious viruses in blood confirmed by plaque assay, but mice expressing human cytokines showed higher viremia compared to conventional NSG mice. The hu-SGM3-PBMCs model developed lethal infections, showing a significant increase in viremia and clinical signs. A detectable human cytokine response was observed in all the DENV-2-infected humanized mouse models. In conclusion, humanized NOD-derived mouse models expressing human cytokines offer a relevant platform for the study of dengue pathogenesis and antiviral therapies.

## 1. Introduction

Dengue fever, caused by any of the four genetically different types of Dengue virus (known as serotypes DENV 1–4) [1], is a significant global public health problem. It is estimated that more than 390 million cases worldwide occur annually [2], and around 60% of the global population is at risk of infection due to a constant expansion of the mosquito vectors, *Aedes aegypti* and *Aedes albopictus,* influenced by global warming, socioeconomic conditions, and poor vector control [3]. 

Although DENV fever is often an asymptomatic or mild infection, 10% of the cases progress to severe dengue with warning signs that may include severe abdominal pain, nose or gum bleeding, and hematemesis, among others [4]. Additionally, a secondary infection with a different DENV serotype can increase the risk of developing severe dengue by a mechanism known as antibody-dependent enhancement (ADE) [5]. There is no antiviral treatment for DENV infection, and the only licensed vaccine available can be applied only in endemic areas [6,7]. 

The development of new therapies and pre-clinical studies in dengue has been challenged by the lack of a suitable in vivo model able to recapitulate disease signs and biomarkers. Previously, non-human primates (NHP) have been used as models [8,9]. However, although rhesus macaques have shown some clinical signs of infection [10], their cost and supply constraints limit the use of these animal models for new pathogenesis studies and therapy development. Considering these factors, organizations such as the U.S. Food and Drug Administration (FDA) promote the use of alternative models [11]. Among these models, mice deficient in type I IFN immunity have been widely used in dengue research [12,13,14,15]. However, the genetic and immune system variations between mice and humans have hindered studies aimed at understanding unique human immune cell infections and responses using mice [16]. To address these species-specific differences during pre-clinical studies, humanized (hu) mouse models have been developed using immunodeficient mice with xenoengraftment of human hematopoietic stem cells (h-HSC) or peripheral blood mononuclear cells (hPBMCs) [17,18].

The humanized mouse model using the immunodeficient strain NOD.Cg-Prkdc^scid^ Il2rg^tm1Wjl/SzJ^ (NSG) as the backbone has been used for dengue research since 2005 [19]. These models have shown a strain-dependent infection with a constant viremia in blood [20]. In addition, they show mild signs of infection such as a decrease of human platelets and increase in body temperature [19,20,21,22,23,24,25,26], as well as T/B cells, monocyte, and megakaryocyte infection [21,24,25]. Moreover, in a hu-NSG model co-transplanted with human fetal thymus and liver (BLT-NSG), DENV infection induced T cell responses as well as IgM and IgG production [22].

In recent years, new immunodeficient mouse strains have been developed. Among them, NOD-derived strains such as the NSG-SGM3 and NOG-EXL were genetically modified to express human cytokines such as interleukin (IL)-3, granulocyte-macrophage colony-stimulating factor (GM-CSF), and stem cell factor [27,28]. In our previous work, we showed that NSG-SGM3 and NOG-EXL hu-mice xenoengrafted with h-HSC from embryonic cord blood enhanced the development of human CD4+ and CD8+ T cells, monocytes, and megakaryocytes cells when compared to hu-NSG conventional mice [29]. Additionally, xenoengraftment with human peripheral blood mononuclear cells (hPBMCs) has been used for the study of human viral infections such as HIV [30]. However, the impact of additional human cytokines to maintain different immune cell populations and the use of humanized mouse models with hPBMC xenotransplants have not been described in DENV infection. The expression of human cytokines is important for the maintenance of human myeloid and lymphoid cells, which are major targets of DENV. This important feature of human cytokine-expressing NOD-derived models, together with the relative low cost and fast development of hPBMC xenotransplants, may accelerate the development of a hu-mouse model for pre-clinical and basic research in dengue. 

This study aimed to compare four hu-mouse models (hu-NSG, hu-SGM3, hu-EXL, and hu-SGM3-PBMCs) for DENV-2 infection. The first three models (hu-NSG, hu-SGM3, and hu-EXL) were xenotransplanted with HSCs derived from cord blood from a single donor, allowing a direct comparison among strains, and a fourth model was developed using the SGM3 mouse strain xenotransplanted with hPBMCs from a healthy donor.

## 2. Materials and Methods

### 2.1. Cell Lines and Dengue Virus

C6/36 (Aedes albopictus cell line) and BHK-21 were acquired from ATCC and maintained in RPMI medium (Gibco, Thermo Fisher Scientific, Waltham, MA, USA) with FBS 10% (R&D SYSTEMS, Minneapolis, MN, USA) ( at 28 °C (C636) and 37 °C (BHK-21) at 5% CO_2_ in a humidified incubator. Dengue virus 2 (DENV-2) strain New Guinea C was purchased from ATCC. The strains were passed in C6/36 cells and kept frozen at −80 °C in 30% FBS. 

### 2.2. Mice and Xenotransplantation

All animal care and procedures were performed under protocol number 032009, reviewed and approved by the Institutional Animal Care and Use Committee (IACUC) at the University of Maryland School of Medicine. The collection of blood from patients was approved by the Institutional Review Board of the University of Maryland School of Medicine (certificate number HP-00040021). NSG (NSG (NOD.Cg-Prkdc^scid^ Il2rg^tm1Wjl/SzJ^) and NSG-SGM3 (NOD.Cg-Prkdc^scid^ Il2rg^tm1Wjl^ Tg (CMV-IL3,CSF2,KITLG)1Eav/MloySzJ) mice were purchased from Jackson Laboratories (Bar Harbor, ME, USA). NCG (NOD-Prkdc^em26Cd52^Il2rg^em26Cd22^/NjuCrl) mice were purchased from Charles River Laboratories (Rockville, MD, USA), and NOG-EXL (NOD.Cg-Prkdc^scid^ Il2rg^tm1Sug^Tg (SV40/HTLV-IL3,CSF2)10-7Jic/JicTac) mice were purchased from Taconic Biosciences (Germantown, NY, USA). All mice were maintained at the Institute of Human Virology at the University of Maryland School of Medicine, Baltimore, USA, and housed in ventilated micro-isolation cages with autoclaved water and irradiated food in a high-barrier facility under specific pathogen-free conditions. For the xenotransplant, human cord blood cells from an anonymous donor were purchased from LONZA (Rockville, MD, USA). Only one donor batch was used to reconstitute all four hu-mice strains. For the hu-mice hPBMCs model, human PBMCs from a healthy donor (IgM and IgG negative evaluated by microneutralization assay and with a history of exposure to DENV) were used for transplantation.

NSG, NSG-SGM3, and NCG 6–8-week-old mice juveniles were conditioned with 25 mg/kg of busulfan given 24 and 48 h intraperitoneally before a vein injection of 50 μL of hCD34+ with 80,000 cells. For the NSG-SGM3 PBMCs model, 6–8-week-olds were intraperitoneally injected with 3.5 × 10^6^ human PBMCs in 300 μl of PBS 1× without conditioned treatment. Since NOG-EXL mice are IL2rgtm1Sug heterozygous, only females were used in this strain. 

After 14 weeks post-xenotransplant, hu-NSG, hu-SGM3, and hu-EXL mice transplanted with HSC hCD34+ were screened for hCD45 (Clone 2D1), hCD20 (2H7), hCD3 (UCHT1), hCD4 (OKT4), and hCD8 (SK1) cells in peripheral blood. Hu-SGM3 PBMCs mice were screened for the same cell markers three weeks after xenotransplant. Only humanized mice with >5% of hCD45+ in peripheral blood were included in this study (Figure S6).

### 2.3. Dengue Infection of Humanized Mice

Hu-mice were intravenously (I.V.) infected with 1 × 10^7^ PFU/mL of DENV-2 (strain NCG) in 100 μl of viral suspension in the tail vein. Control hu-mice were inoculated with 100 μl of C636 supernatants, and immunodeficient mice without xenotransplant of each strain were infected with 1 × 10^7^ PFU/mL in 100 μl of viral suspension I.V. Clinical signs of infection (weight and temperature) were assessed on days 0, 2, 4, 9, 11, 14, 17, and 21 p.i. Temperature was measured via rectal using a Type J/K/T thermocouple thermometer with RET-3 rectal probe (Kent Scientific^®^, Torrington, CT, USA). Mice were anesthetized via isoflurane inhalation, and approximately 50 μl of blood was obtained via retroorbital bleed to quantify viremia. Blood was transferred to Greiner Bio One MiniCollect tube Levander 0.5 mL K2EDTA before centrifugation at 300× *g* for 3 min to separate the plasma. Samples were stored at -80 °C until RNA extraction and qRT-PCR were performed. On days 0, 3, 5, and 7 post-infection, blood was collected via retroorbital bleed to perform platelet flow cytometry. 

### 2.4. Quantification of Viral RNA 

Viral RNA was extracted from 10 µL of plasma samples from hu-NSG, hu-EXL, hu-SGM3, and hu-SGM3 PBMCs mice using the QiAmp^TM^ RNA viral Mini kit (Qiagen^®^, Germantown, MD, USA) according to the manufacturer’s instructions. Viral RNA was quantified via one-step qRT-PCR TaqMan^TM^ Fast virus 1-steo multiplex master mix (Applied Biosystem^TM^, Thermo Fisher Scientific, Waltham, MA, USA) using the manufacturer’s instructions. Moreover, 5 µL of RNA were used per reaction. Primers, probe sequences, and cycling conditions are described in Table 1 and Table 2, respectively. This assay has a detection limit of 9.8 × 10^2^ genome eq/mL for DENV-2 [31]. An absolute standard curve using quantitative synthetic RNA from dengue virus type 2 (VR-3229SD^TM^) from ATCC was used to determinate the concentration of viral RNA genome copies in samples. All the samples were run on a QuantStudio^TM^ 3 Real-Time PCR System (Applied Biosystems^TM^).

### 2.5. Cytometric Bead Array (CBA) Analysis 

An aliquot of 10 µL of plasma from three infected humanized mice and one control at day 0, day +7, and day +21 post-DENV-2 infection were collected and tested for human IL-6 (#558276 BD Biosciences, Franklin Lakes, NJ, USA), IFN-γ (#558269 BD Biosciences), IFN-α (#560379 BD Biosciences), TNF (#560112 BD Biosciences), IL-12p70 (#558283 BD Biosciences), IL-10 (#558274 BD Biosciences), and IP-10 (#558280 BD Biosciences) using the CBA array kit (#558264 BD Biosciences). Standard curves using human proteins were used to measure cytokine concentration. Data were analyzed with FlowJo Version 10. 

### 2.6. Detection of Platelets

For platelet detection in the hu-mouse models, 30 μL of peripheral blood were collected in 600 μL of a megakaryocyte buffer (0.1 mM theophylline (Sigma, Burlington, NJ, USA), 15 mM sodium citrate (Sigma), and 1% BSA (Sigma) in PBS 1× sterile), followed by centrifugation at 100× *g* for 15 min at room temperature (RT). Then, the supernatant was centrifuged at 1000× *g* for 10 min and subsequently discarded, and the pellet was collected [32]. The platelets were cautiously resuspended in PBS 1× to avoid undesired activation and then stained with anti-human hCD41+ (HIP8), CD42a (GR-P), CD42b (HIP1), and CD62P (AK4) [33].

### 2.7. Indirect Plaque Assay Titration

Serum samples from mice on day 7 and endpoint were collected by terminal bleeding, and the virus was isolated in one passage on C6/36 for seven days. Subsequently, the supernatants were used for quantification by plaque assay with BHK-12 cells [34]. Additionally, RT-qPCR (Previously described) was used to quantify the genome copies and confirm the presence of the virus in isolation samples that did not develop plaques. 

### 2.8. Statistical Analysis

Data from the experiments were presented as the mean plus standard deviation (SD) and graphed using GraphPad Prism Version 8. All data were subjected to a normality test using a Shapiro–Wilk test, after which the respective statistic tests were selected. Kaplan–Meier and log-rank (Mantel–Cox) tests were used for the survival curve; all data were subjected to a normality test using a Shapiro–Wilk test, after which a nonparametric Kruskal–Wallis test followed by a Dunn test was used. A *p*-value < 0.05 was considered statistically significant. 

## 3. Results

### 3.1. DENV-2 Infection May Lead to Lethality in Hu-Mice Xenotransplanted with Human CD34+ Hematopoietic Stem Cells

**Figure 1:**
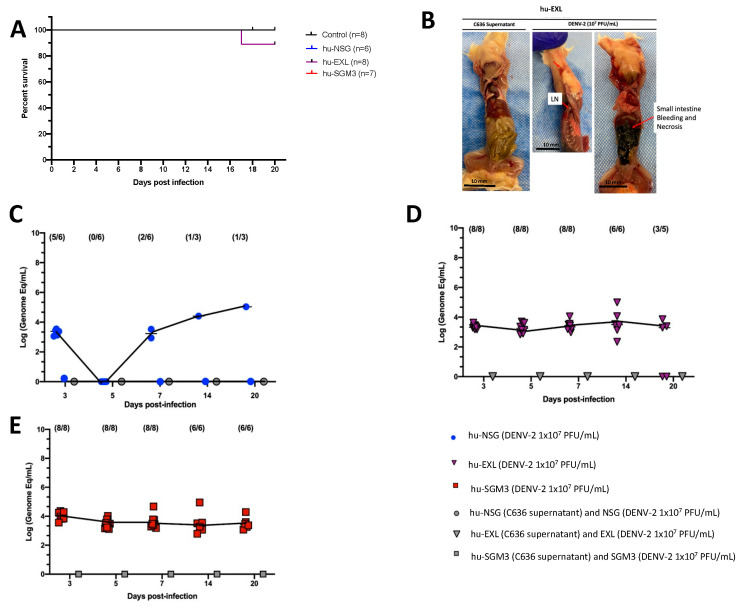
Comparison of DENV−2 disease and replication in hu-NSG, hu-EXL, and hu-SGM3 mouse models. (**A**) Kaplan–Meier survival curves depicting the outcomes for hu-NSG (*n* = 6), hu-EXL (*n* = 8), and hu-SGM3 (*n* = 7) mice infected with 10^7^ PFU/mL via intravenous route. Two controls for each mouse model were used. Mice were closely monitored over a 20-day period. (**B**) Necropsy findings illustrating a hu-EXL-infected mouse’s demise on the 17th day post-infection, revealing bleeding and necrosis of the small intestine with the presence of lymph nodes (LN). (**C**) Viral kinetics in the hu-NSG model after xenotransplantation with CD34+ HSC. hu-NSG (DENV−2 1 × 10^7^ PFU) are infected mice; hu-NSG (C636 supernatant) are control mice, and NSG (DENV−2 1 × 10^7^ PFU) are non-humanized mice inoculated with DENV. (**D**) Viral kinetics in the hu-EXL model expressing human cytokines and xenotransplanted with CD34+ HSC. hu-EXL (DENV−2 1 × 10^7^ PFU) are infected mice; hu-EXL (C636 supernatant) are control mice, and EXL (DENV−2 1 × 10^7^ PFU) are non-humanized mice inoculated with DENV. (**E**) Viral kinetics in the hu-SGM3 model expressing human cytokines and xenotransplanted with CD34+ HSC. hu-SGM3 (DENV−2 1 × 10^7^ PFU) are infected mice; hu-SGM3 (C636 supernatant) are control mice, and SGM3 (DENV−2 1 × 10^7^ PFU) are non-humanized mice inoculated with DENV. At the upper section of each diagram, the count of positive samples is indicated for the total serum samples analyzed on each respective day. No statistical difference was observed in the survival curves between models (log-rank (Mantel–Cox) test *p* > 0.05) or viremia between hu-EXL and hu-SGM3 (Kruskal–Wallis *p* > 0.05).

The DENV−2 New Guinea C strain was selected for this study as a reference strain for its easy access and frequent use in DENV research. Xenotransplanted hu-NSG, hu-EXL, and hu-SGM3 mouse strains expressing human IL-3, GM-CSF, and stem cell factor were intravenously inoculated with a dose of 1 × 10^7^ Plaque-forming units (PFU/mL). All mice survived the challenge with no statistical difference in the survival curve between these models. No mice in any of the models exhibited clinical signs of infection, including weight loss, increased body temperature, or observable discomfort such as hunchbacked posture or piloerection (Figures S1 and S2). However, one death at day 17 post-infection (p.i.) in the hu-EXL group was observed (Figure 1A). Upon necropsy of the only deceased hu-EXL mouse, noticeable intra-abdominal hemorrhage and necrotic changes were observed in the small intestine. However, no such changes were present in the other infected mice (Figure 1B). 

Considering that a reduction in platelet count is a prominent clinical manifestation of dengue infection in humans [35], we evaluated the dynamics of human platelets in the humanized mouse models by flow cytometry. We observed a trend for decreased frequencies of human platelets (CD41+ CD42a+ CD42b+) on day 3 in hu-NSG, days 3 and 5 in hu-EXL, and days 5 and 7 in hu-SGM3, coupled with an activated phenotype (CD62P+) across all humanized mouse models that received DENV−2 challenge relative to non-infected controls (Figure S3).

### 3.2. More Sustained DENV-2 Replication in Hu-Mouse Models Expressing Human Cytokines Relative to Conventional NSG Mice

We compared the level of DENV-2 replication in conventional hu-NSG and hu-mice expressing human cytokines (hu-EXL and hu-SGM3). Viral load was assessed in sera at 3, 5, 7, 14, and 20 days (p.i.). To ensure uniformity across the models, the same viral stock and source of CD34+ cells for the xenotransplant were employed. In line with prior reports, it was observed that DENV-2-infected hu-NSG mice exhibited heterogeneous viremia dynamics along time [24]. As such, at 3 days p.i., viremia was detected in 5 out of 6 infected hu-NSG mice, although this number decreased to 0 out of 6 by day 5 p.i. By day 7 p.i., this proportion increased to two out of six infected mice (Figure 1C). In contrast, all hu-mice expressing human cytokines (hu-EXL and hu-SGM3) showed consistent detectable viremia from day 3 through day 20 p.i., and no statistical difference between the viremia of hu-EXL and hu-SGM3 was observed (Figure 1D,E). 

To confirm that the level of xenoengraftment did not influence the development of stable viremia and that viremia observed in hu-NSG was not restricted to this specific NOD-derived strain, we evaluated a group of hu-SGM3 and hu-NCG (CD34+) mice with low and high xenoengraftment (Figure S4). The NCG mice were selected since they are a conventional NOD-derived strain and exhibit a comparable degree of immunodeficiency to NSG mice [36]. We did not find a direct correlation between the level of human cells in these models and the degree of viremia (Figure S4). We observed that even ~3% of human cells in peripheral blood are enough to develop a stable and constant viremia in the hu-SGM3 CD34+ (Figure S4C–E). However, as expected, NSG, NCG, SGM3, and EXL strains lacking xenotransplants did not develop viral replication upon DENV-2 challenge (Figure 1). Additionally, as we described for the hu-NSG mice, the viremia in the hu-NCG mice was heterogeneous in the frequency of mice with detectable viruses and completely disappeared by day 14 p.i. (Figure S4G). 

**Figure 2:**
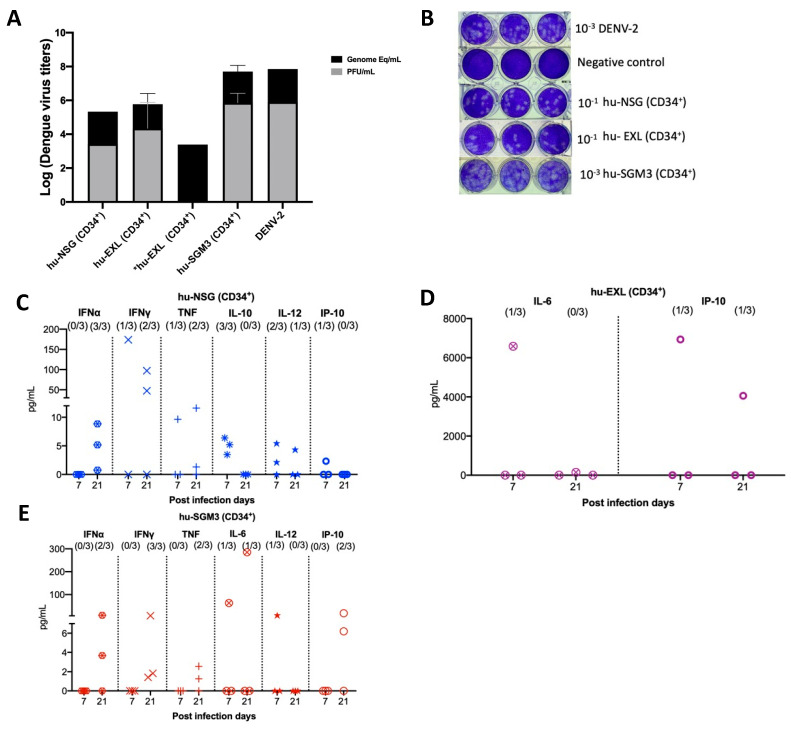
Identification of infectious virus in RT-qPCR-positive serum samples from hu-mouse models (CD43+) and cytokine profile in hu-mice models infected with DENV−2 at 7- and 20-days post-infection. (**A**) Relation between plaque formation units (plaque assay) and genome equivalent (RT-qPCR) copies per mL observed in supernatants derived from the indirect plaque assay (n = 3); DENV−2 propagated in C636 was used as a control; *hu-EXL sample did not develop plaques yet still yielded a positive result when subjected to RT-qPCR. (**B**) Representative plaque assay images of samples from each model; DENV−2 propagated in C636 was used as a positive control, and RPMI media was used as a negative control. Human cytokine profile detected in (**C**) hu-NSG (CD34^+^), (**D**) hu-EXL (CD34^+^), and (**E**) hu-SGM3 (CD34^+^). Each plot displays data from three individual mice within their respective groups; values from control mice inoculated with C636 supernatant control sera were subtracted. At the upper section of each diagram, the count of positive samples is indicated for the total serum samples analyzed on each respective day.

We confirmed the RT-qPCR viremia results of detectable samples by an indirect plaque assay, which measures the level of infectious virus. We found that infectious particles associated with viral replication were indeed present in nearly all serum samples that had tested positive via RT-qPCR across the hu-NSG, hu-EXL, and hu-SGM3 (Figure 2A,B). Notably, one of the supernatants from an isolated sample did not develop plaques yet still yielded a positive result when subjected to RT-qPCR (Figure 2A). This latter result suggests that the virus requires a certain threshold of genome-equivalent copies before plaque formation can occur or may be related to the presence of a high number of defective particles in this mouse. 

### 3.3. Detectable Human Cytokine Response in DENV−2-Infected Humanized Mice CD34+ 

To delineate the profile of human cytokines during DENV−2 infection in the previously described models, a panel of human cytokines associated with dengue infection was analyzed at days 7 and 21 post-infection (IL-6, IFN-γ, IFN-α, TNF, IL-12p70, IL-10, and IP-10). In all the hCD34+ humanized models, we observed low-to-undetectable levels of human cytokines with a heterogeneous pattern of detection. In the case of Hu-NSG (CD34+), seven cytokines were detected, with a predominance of the IFN-α response (3/3 mouse) and IFN-γ (2/3 mouse) by day 21 post-infection (Figure 2C). In the Hu-EXL (CD34+) model, we only detected the proinflammatory cytokines IL-6 and IP-10 (1/3 mouse) (Figure 2D). Finally, in the Hu-SGM3 (CD34+) model, six cytokines were detected, including IFN-α (2/3 mouse) and IFN-γ (3/3 mouse) by day 21 post-infection (Figure 2E). 

### 3.4. DENV-2 Infection in Hu-SGM3 Xenotransplanted with Human PBMCs

**Figure 3:**
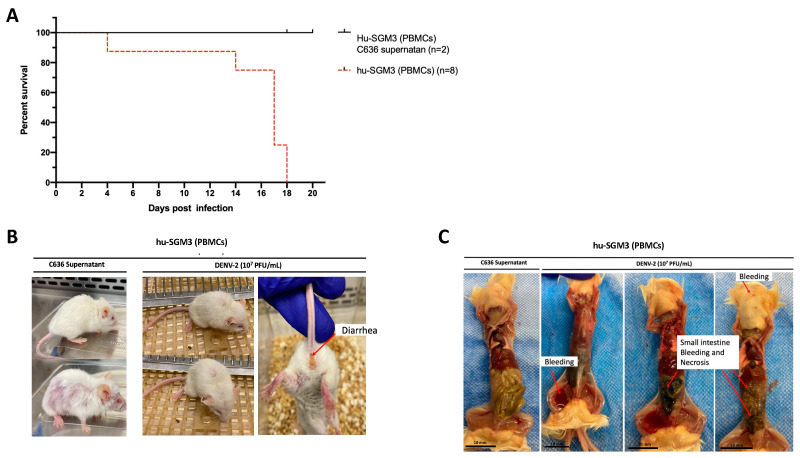
Assessment of DENV−2 disease in hu-SGM3 (PBMCs), mouse model. (**A**) Kaplan–Meier survival curves depicting the outcomes for hu-SGM3 PBMCs (n = 8) mice infected with 10^7^ PFU via intravenous route. Two controls for each mouse model were used. Mice were closely monitored over a 20-day period. (**B**) Clinical manifestations captured in hu-SGM3 PBMC mice infected with DENV-2 at 10^7^ PFU/mL via the intravenous route on day 18. Infected mice exhibit diarrhea and a hunchbacked posture, while the controls displayed hair loss due to Graft versus Host Disease (GvHD). (**C**) Representative necropsy findings from hu-SGM3 PBMC mice (five out of eight mice), uncovering internal bleeding and small intestine necrosis. Control (C636 Supernatant) in C images corresponds to the mouse displaying the GvHD phenotype. The survival curves between hu-SGM3 (PBMCs), hu-NSG (CD34+), hu-EXL (CD34+), and hu-SGM3 (CD34+) showed a statistically significant difference (*p* < 0.001) log-rank (Mantel–Cox) test.

The humanized mouse model was established through xenotransplantation of human PBMCs obtained from a healthy donor with a prior history of DENV infection (Figure S5). Upon infecting hu-SGM3 PBMCs with DENV−2 at a concentration of 1 × 10^7^ PFU/mL via the intravenous route, distinctive clinical manifestations were observed. All Hu-SGM3 hPBMCs-infected mice died by day 18 post-infection compared to controls, whose survival was not affected during this study (Figure 3A). This difference in infection outcome was statistically significant when comparing survival curves among hu-SGM3 (PBMCs), hu-NSG (CD34+), hu-EXL (CD34+), and hu-SGM3 (CD34+) (*p* < 0.001). The infected mice exhibited symptoms such as diarrhea and an arched posture (Figure 3B). The control group experienced hair loss attributed to Graft versus Host Disease (GvHD) but did not develop diarrhea or arched posture (Figure 3B). Necropsy from the hu-SGM3 hPBMCs mice (five out of eight mice) infected with DENV−2, but not uninfected mice, revealed signs of internal bleeding and necrosis in the small intestine (Figure 3C). 

**Figure 4:**
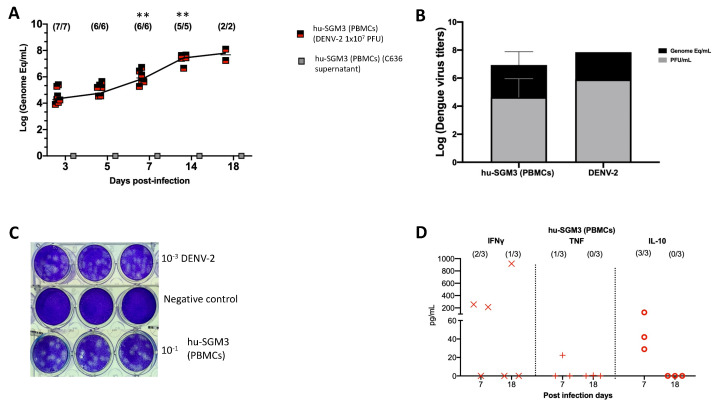
Dynamics of DENV−2 infection in the hu-SGM3 PBMCs model. Blood samples were collected, and subsequent serum samples were used for viremia and human cytokine assessment. (**A**) Viral kinetics in the hu-SGM3 PBMCs model. hu-SGM3 (PBMCs) (DENV−2 1 × 10^7^ PFU) are infected mice, and hu-SGM3 (PBMCs) (C636 supernatant) are control mice. (**B**) Relation between plaque formation units (plaque assay) and genome equivalent (RT-qPCR) copies per mL observed in supernatants derived from the indirect plaque assay (n = 3); DENV−2 propagated in C636 was used as a control. (**C**) Representative plaque assay images of indirect plaque assay, DENV−2 propagated in C636 were used as a positive control, and RPMI media was used as a negative control. (**D**) human cytokine profile in hu-SGM3 PBMCs model infected with DENV−2 at 7- and 18-days post-infection. Values from hu-SGM3 PBMCs inoculated with C636 supernatant control sera were subtracted. At the upper section of each diagram, the count of positive samples is indicated for the total serum samples analyzed on each respective day. ** *p* < 0.001 (Dunn test) when compared with the hu-EXL (CD34+) and hu-SGM3 (CD34+).

Consistent with these clinical findings, the hu-SGM3 PBMCs model exhibited statistically significant high viral titers of DENV−2 on days 7, 14, and 18 (*p* < 0.001) (Figure 4A), being 2–4 log higher than in the hCD34+ humanized models (Figure 1). As expected, we did not detect viremia in hu-SGM3 hPBMCs challenged with C636 supernatant alone (Figure 4A). The presence of infectious particles in DENV−2-infected mice was further corroborated by the indirect plaque assay (Figure 4B,C). Finally, when the human cytokine profile was analyzed in the Hu-SGM3-PBMCs model, there was a detection of high concentrations of IFN-γ (2/3 mice) and the anti-inflammatory cytokine IL-10 (3/3 mice) by day 7 post-infection (Figure 4D).

## 4. Discussion

In this study, we characterized three humanized mouse models for the study of DENV−2 infection, using immunodeficient mice expressing human cytokines and xenografted with hCD34+ cells from cord blood or adult PBMCs. All the models developed viremia, with no clinical signs in the case of hu-EXL and hu-SGM3 xenografted with hCD34 cells. However, the hu-SGM3-PBMCs model developed an exponential and deadly viremia in infected mice. 

We used the DENV-2 New Guinea C strain, commonly employed for in vitro studies due to its non-adapted nature in mice and wide accessibility [37]. Consistent with our data, previous studies using hu-NSG mouse models infected with this viral strain did not observe clinical symptoms or sustained viremia [24]. However, our plaque assay revealed the presence of infectious particles in some mice, indicating that the DENV-2 New Guinea C strain can replicate in conventional NSG or NCG strains but with low efficiency. More work is needed to understand why the New Guinea C strain did not efficiently replicate in the hu-NSG and hu-NCG even though these mouse strains develop human monocytes [29] and why there was not a correlation between viremia and the level of xenoengraftment in this model. 

Our study demonstrates that humanized mouse models expressing human cytokines improve the detection of viremia during infection. Indeed, the hu-EXL and hu-SGM3 models displayed a consistent and detectable viremia from the onset of infection until day 20 post-infection. Furthermore, a trend for an activated platelet phenotype was seen in the hu-SGM3 model, resembling DENV infection in humans [38]. To improve the study of platelets during DENV infection in this humanized model, murine macrophage depletion could be employed, as previously reported [39]. 

As observed for conventional hu-NSG, in the hu-EXL and hu-SGM3 mouse models, we did not find any correlation between viremia and the level of engraftment. However, there was a minimum amount of human cells required to develop a stable dengue viremia in the mice. This implies that the replication efficiency of the virus in these models may be derived not only by the presence of human cells but could also be influenced by factors such as cell receptor abundance [40]. In this regard, GM-CSF promotes the expression of CD14 and mannose receptor (CD206) in monocytes and macrophages, as well as DC-SIGN expression in dendritic cells [41,42,43], all of which are receptors involved in DENV cell entry [44,45,46]. The expression of human GM-CSF in the hu-SGM3 and hu-EXL mice thus may facilitate DENV infection by increasing virus receptor abundance, even in the context of a low level of engraftment in some animals.

Despite high levels of viral replication, we did not observe clinical signs of infection (such as weight loss or fever) in any of the CD34+ hu-mice models. However, we found the production of some pyrogenic cytokines, such as tumor necrosis factor (TNF) in hu-NSG and hu-SGM3, and IL-6 in hu-SGM3 and hu-EXL xenografted with hCD34+ cells, as reported previously [21]. It is crucial to consider two factors when analyzing the immune response in these xenograft models. First, human cytokine responses could have a limited effect in the mouse given the potential lack of ligand-receptor interaction between both species [47], thus limiting the activation of the IL-6-COX2-PGE2 axis responsible for the fever in mice [48]. Additionally, the GvHD inherent to xenoengraftment in this model introduces a background in cytokine responses that require normalization and vary between models [29]. In this study, although a Th1 antiviral response to dengue infection characterized by the production of cytokines such as IFN-α, IFN-γ, and TNF was observed in hu-NSG (CD34+), hu-SGM3 (CD34+), and hu-SGM3 (PBMCs) [49,50], their concentrations were relatively low. This could be explained by a possible tissue-specific response pattern in this model and the immune modulation characteristics of the New Guinea C strain, which has been reported to inhibit the phosphorylation of STAT molecular signals, thereby impacting the antiviral response [51]. Nevertheless, further investigation into these factors is warranted to better understand their implications in the model’s immune response dynamics. 

We also evaluated the hu-SGM3 model xenografted with human PBMCs. This model not only reduces the humanization time from 14 weeks to 3 weeks compared to xenoengraftments with the HSC CD34+, but also eliminates the need for a conditioning regimen before human cell infusion. Thus, this model may be a more accessible humanized mouse model for dengue research. Beyond these benefits, we observed that the hu-SGM3 PBMCs dengue model exhibited higher levels of viral replication than the other humanized models described. Likely, the presence of human monocytes supported by human cytokine expression in SGM3 mice contributed to the sustained viremia observed in this model [29]. Additionally, the increasing viral titers up to day 18 may be a consequence of an increase in viral replication in organs such as the spleen and liver, as previously reported in a similar mouse model [52]. Inflammatory cytokines may have also contributed to viral burden in this model. Interestingly, in contrast to the CD34+ humanized mouse models, the hu-SGM3 PBMCs dengue model showed weight loss, diarrhea, and gastrointestinal bleeding in some animals, indicating that this model can recapitulate some clinical signs of dengue. Gastrointestinal tract inflammation and severe tissue damage with neutrophils and monocyte infiltration during DENV in ifnar1^-/-^ mice have been reported [53]. In the case of mice xenoengrafted with human PBMCs, the most prevalent population is T cells, which can infiltrate mouse spleen, liver, and gut [30,54]. Although gut-infiltrating immune cells were not characterized in this work, the presence of human monocytes and T cells, along with an inflammatory cytokine environment, may have contributed to tissue damage.

## 5. Conclusions

Here we compared several humanized mouse models for DENV-2 infection, each one with several advantages and limitations (Table 3). Our results indicate that the expression of human cytokines in immunodeficient mice (EXL and SGM3 strains) promotes DENV-2 replication. In addition, the hu-SGM3 PBMCs model recapitulates important features of human DENV infection, such as high viremia, cytokine production, and clinical signs of disease. Thus, these models constitute a new tool for the analysis of the in vivo dynamic of DENV infection for future pathogenesis and therapy studies.

## Figures and Tables

**Table 1:** Primers used to detect viremia in humanized mice models.

Serotype	Forward Primer	Reverse Primer	µM Primers	Probe	µM Probe
**DENV**−**2**	CAGGCTATGGCACYGTCACGAT	CCATYTGCAGCARCACCATCTC	0.2	5′-/56-FMA/CTCYCCRAG/ZEN/AACGGGCCTCGACTTCAA/3IABkFQ/-3´	0.18

**Table 2:** RT-qPCR machine set up.

Step	Sub-Step	Temperature (°C)	Time (s)	Cycles
Reverse transcription	-	50	300	1
Reverse transcription inactivation	-	95	20	1
Amplification	Denaturation	95	15	45
	Annealing and extension	60	60	

**Table 3:** Advantages and limitations for the described humanized mouse models of dengue.

Humanized Mouse Model	Estimated Time Frame for Development	Advantages	Limitations
Conventional hu-NSG and hu-NCG xenografted with hCD34+ cells	14 weeks	Mice strains are more accessible. Slow development of GVHD. Allow the study of innate immunity.	Conditioning is needed for the xenotransplant. HSC CD34+ are difficult to obtain. Immune response to GVHD. Restricted to some dengue virus strains Do not develop clinical signs.
hu-EXL and hu-SGM3 xenografted with hCD34+ cells	14 weeks	Slow development of GVHD Suitable model to study human platelets during infection. Sustained dengue viremia in blood. Allow the study of innate immunity.	Conditioning is needed for the xenotransplant. HSC CD34+ are difficult to obtain. Immune response to GVHD. Do not develop clinical signs.
hu-SGM3 xenografted with human PBMCs	3 weeks	No conditioning is needed for the xenotransplant. Higher and exponential viraemia. Clinical signs of infection. Gastrointestinal compromise.	Limited human innate immunity. Fast GVHD development. Immune response to GVHD. Does not allow to study human platelets. Possible inter-donor variability.

## Data Availability

No new data were created or analyzed in this study. Data sharing is not applicable.

## Supplementary Materials

The following supporting information can be downloaded at: https://www.mdpi.com/article/10.3390/pathogens13080639/s1. Figure S1. Changes in the temperature with respect to the day before DENV-2 infection or C636 supernatant inoculation measured intrarectally in the indicated time points for hu-NSG (A), hu-EXL (B), hu-SGM3 (C), and hu-SGM3 (PBMCs) (D); Figure S2. Body weight changes with respect to the day before DENV-2 infection or C636 supernatant inoculation in the indicated time points for hu-NSG (A), hu-EXL (B), hu-SGM3 (C), and hu-SGM3 (PBMCs) (D); Figure S3. Human platelets (CD41+, CD42+, and CD24b+) percentages and activation profile (CD62P+) in humanized mouse models evaluated by flow cytometry in the indicated time points for hu-NSG (A), hu-EXL (B), and hu-SGM3 (C) xenografted with hCD34+ cells; Figure S4. Level of engraftment and viremia develop in Hu-NCG and Hu-SGM3 xenoengrafted with HSC CD34+; Figure S5. Microneutralization assay from the donor used in the hu-SGM3 (PBMCs) model; Figure S6. Levels of xenoengraftmen (human CD45%) in peripheral blood of humanized mouse models; Figure S7. Human cytokine profile including the GvHD background of (A) hu-NSG 62 (CD34+), (B) hu-EXL (CD34+), (C) hu-SGM3 (CD34+), and (D) hu-SGM3 PBMCs model 63 infected with DENV-2 at 7- and 18-days post-infection. At the upper section of each 64 diagram, the count of positive samples over the background is indicated for the total 65 serum samples analyzed on each respective day. Black dots are the mouse’s background 66 representing the average value of controls (n = 2).
