# Supplementary material for: Evaluation of Four Humanized NOD-Derived Mouse Models for Dengue Virus-2 Infection
Source: Pathogens. 2024 Jul 30;13(8):639. doi: 10.3390/pathogens13080639 (PMC11357684; doi:10.3390/pathogens13080639)
Supplement: Supplementary file 1 [file pathogens-13-00639-s001.zip › pathogens-3101751-supplementary.docx]

**Supplementary Materials:**

**
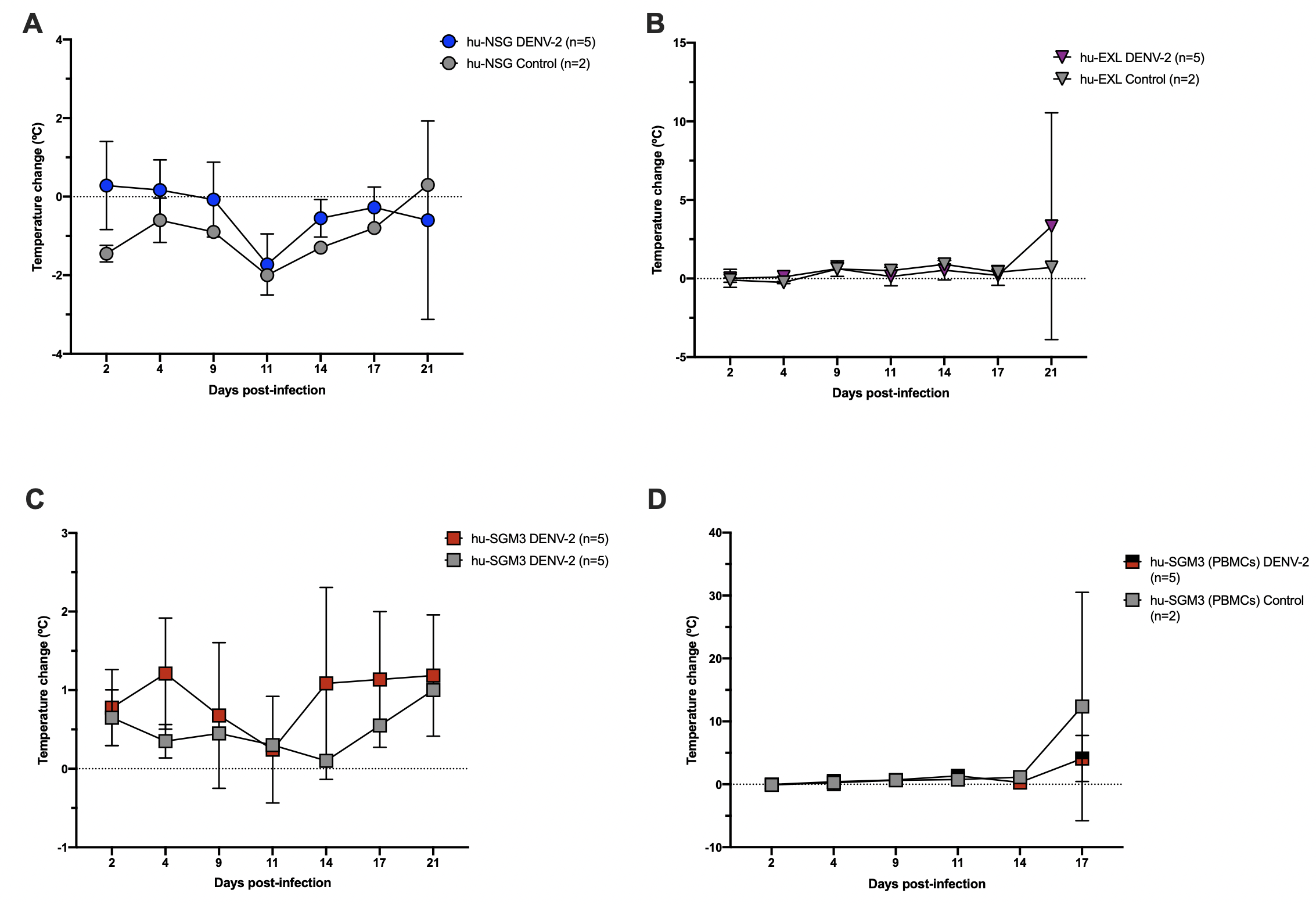
**

**Figure S1.** Changes in the temperature with respect to the day before DENV-2 infection or C636 supernatant inoculation measured intrarectally in the indicated time points for hu-NSG (A), hu-EXL (B), hu-SGM3 (C) and hu-SGM3 (PBMCs) (D).


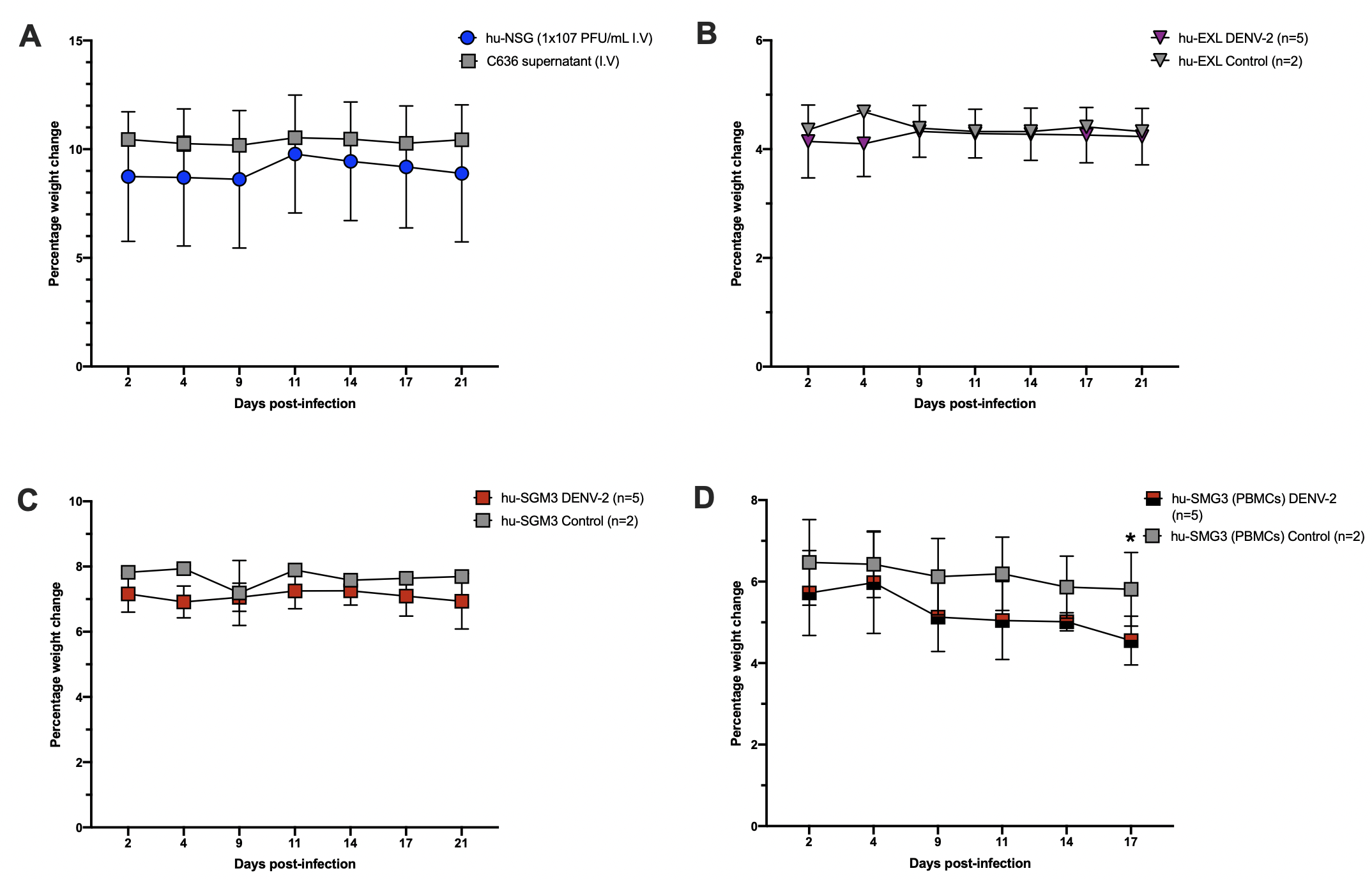


**Figure S2.** Body weight changes with respect to the day before DENV-2 infection or C636 supernatant inoculation in the indicated time points for hu-NSG (A), hu-EXL (B), hu-SGM3 (C) and hu-SGM3 (PBMCs) (D). *p<0.05 Mann-Whitney test


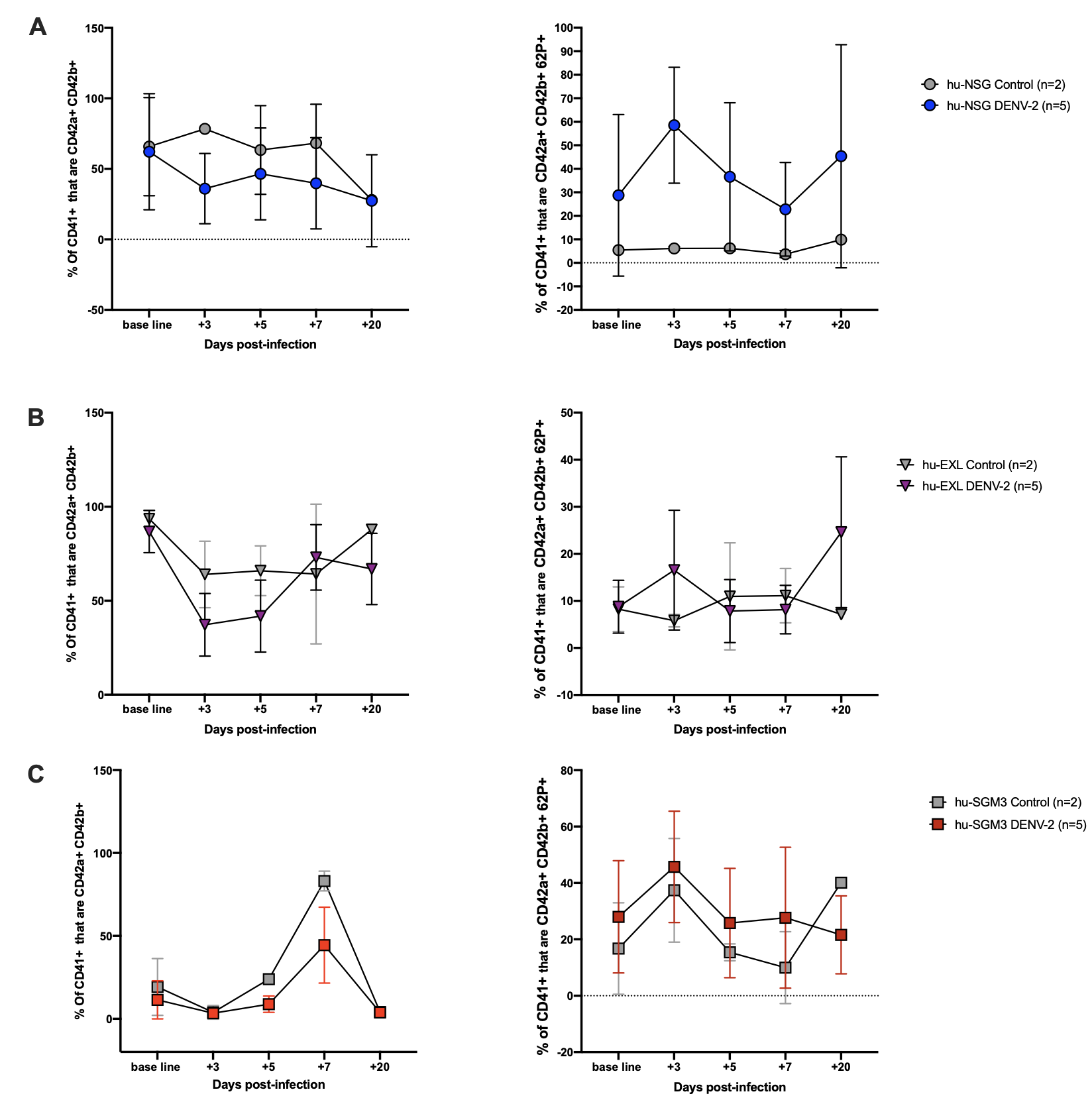


**Figure S3.** Human platelets (CD41^+^, CD42^+^ and CD24b^+^) percentages and activation profile (CD62P^+^) in humanized mice models evaluated by flow cytometry in the indicated time points for hu-NSG (A), hu-EXL (B) and hu-SGM3 (C) xenografted with hCD34+ cells.

**Figure S4.** Level of engraftment and viremia develop in Hu-NCG and Hu-SGM3 xenoengrafted with HSC CD34^+^. Six hu-SGM3 and 8 hu-NCG mice were infected with 1x10^7^ PFU/ml and viremia was followed for 20 days. Level of xenoengraftment of Hu-SGM3 mice infected with DENV-2 New guinea C strain (A). Viremia in plasma measured by RT-qPCR at the indicated time points; the number of mice with detectable viremia/total mice is showed in each time point (B). Nonparametric Spearman correlation between the initial level of engraftment and viremia in Hu-SGM3 at day 7 (C), 14 (D) and 20 (-E) days post-infection. Doted lines indicate the minimum engraftment level in which viremia was detected in plasma for each time point. Level of xenoengraftment of Hu-NCG mice infected with DENV-2 New guinea C strain (F). Viremia in plasma measured by RT-qPCR at the indicated time points; the number of mice with detectable viremia/total mice is showed in each time point (G). Nonparametric spearmen correlation between initial level of engraftment and viremia in Hu-NCG at day 7 (H), 14 (I) and 20 (J) days post-infection.


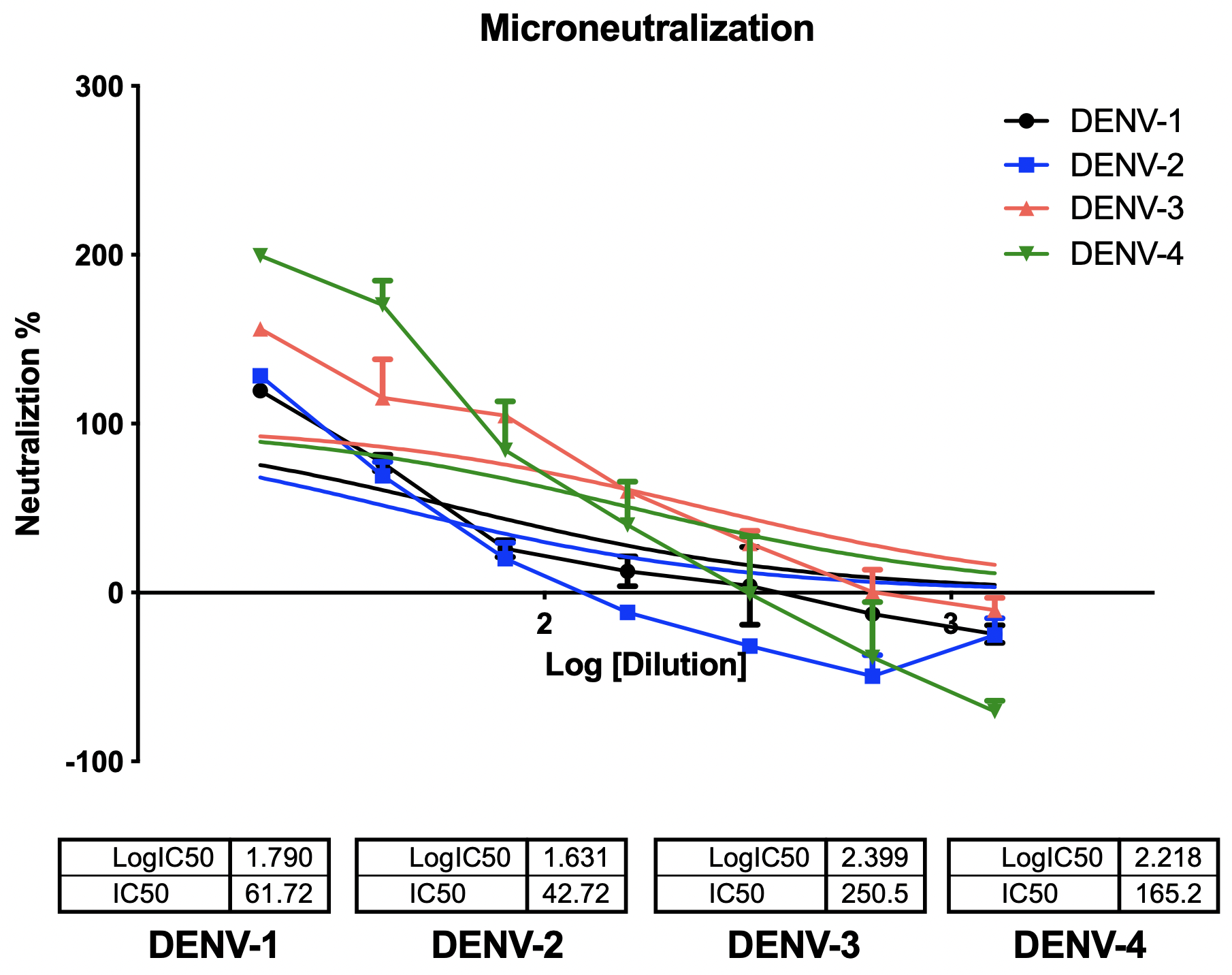


**Figure S5.** Microneutralization assay from the donor used in the hu-SGM3 (PBMCs) Model. The percentage of neutralization of infection in BHK-21 cells post viral infection at MOI 0.5 and IC50 was calculated for each DENV serotype in each serum dilution.


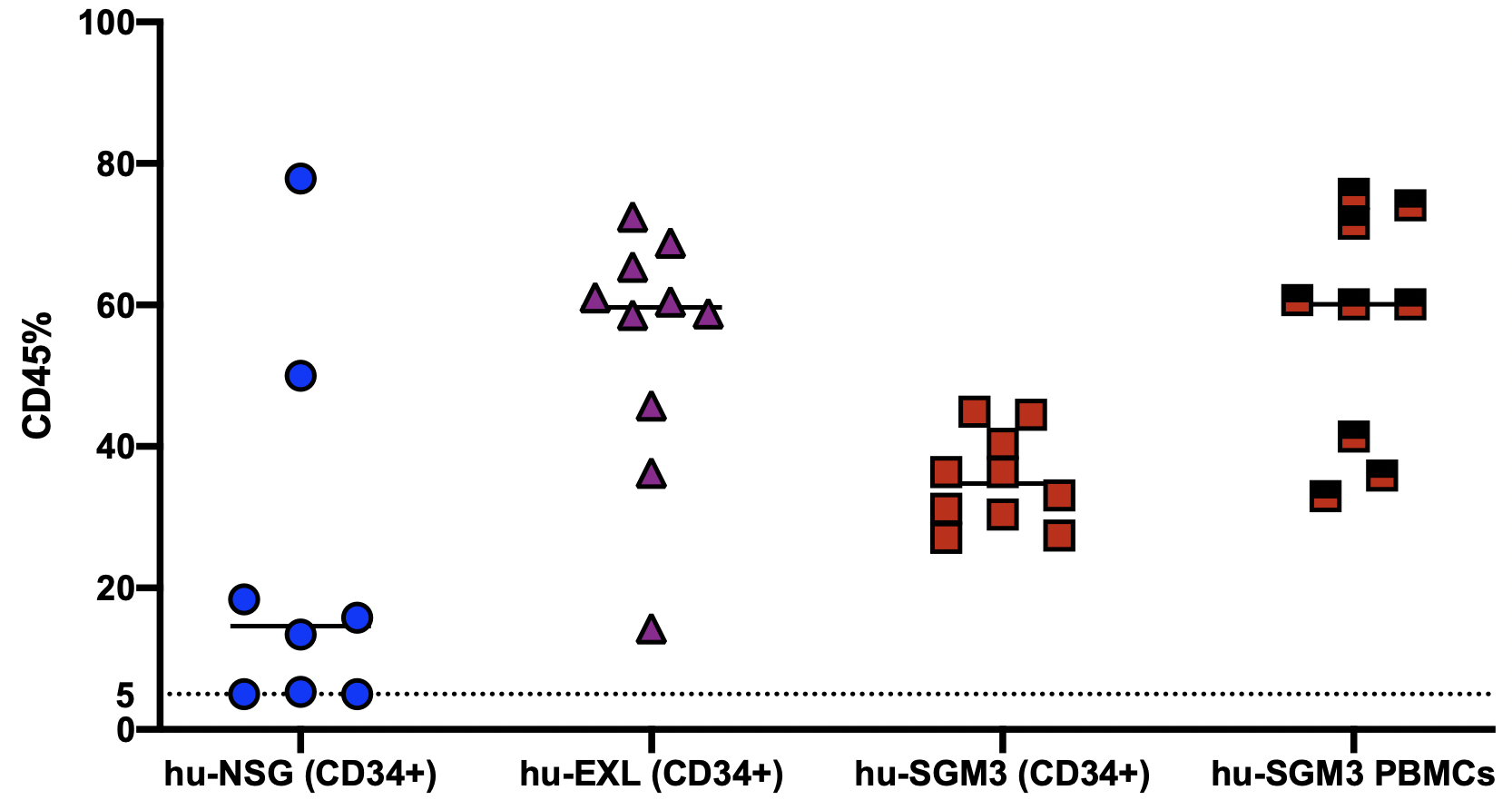


**Figure S6.** Levels of Xenoengraftmen (human CD45% ) in peripheral blood of humanized mouse models. Humanized mouse hu-NSG, hu-SGM3, hu-EXL mice transplanted with HSC hCD34+ After 14 weeks post xenotransplant, and hu-SGM3 PBMCs mice three weeks after xenotransplant with human PBMCs, screened for the human CD45^+^ cells. The dotted line indicates the selection threshold used in the study (5%).

**Figure S7.** Human cytokine profile including the GvHD background of (A) hu-NSG (CD34^+^), (B) hu-EXL (CD34^+^), (C) hu-SGM3 (CD34^+^), and (D) hu-SGM3 PBMCs model infected with DENV-2 at 7- and 18-days post-infection. At the upper section of each diagram, the count of positive samples over the background is indicated for the total serum samples analyzed on each respective day. Black dots are the mouse's background representing the average value of controls (n=2).
